# Spontaneous intraperitoneal rupture of hepatic hydatid cyst with biliary peritonitis: a case report

**DOI:** 10.4076/1757-1626-2-6511

**Published:** 2009-08-11

**Authors:** Sukanta Ray, Kausik Das

**Affiliations:** 1 Division of Surgical Gastroenterology, School of Digestive and Liver DiseasesI.P.G.M.E.R, Kolkata 700020, West BengalIndia; 2 Hepatology, School of Digestive and Liver DiseasesI.P.G.M.E.R, Kolkata 700020, West BengalIndia

## Abstract

**Introduction:**

Spontaneous intraperitoneal rupture with biliary peritonitis in a case of hepatic hydatid cyst is extremely rare but serious complication. It is a surgical emergency with high morbidity and mortality rates. Very few cases have been reported in the literature.

**Case presentation:**

We report an unusual case of a biliary peritonitis due to spontaneous rupture of hepatic hydatid cyst in a male patient of 34 years of age. He presented with acute peritonitis. Contrast enhanced computed tomography 2 days prior to laparotomy showed a dumbbell shaped hydatid cyst of right lobe of the liver with perihepatic fluid collection. At operation 1.5 L bile was found in the peritoneal cavity with rupture of the anterior wall of the cyst and large cystobiliary communication. He was managed with deroofing of the cyst, cholecystectomy, and placement of T tube in the cystobiliary communication and in the extrahepatic bile duct. He developed biliary fistula which was closed over a period of 34 days with conservative therapy. At 6 months follow up patient is well and free of recurrence.

**Conclusion:**

Though rare, ruptured hydatid cyst should be considered in the differential diagnosis of acute abdomen in a patient residing in the endemic zone.

## Introduction

Intraperitoneal rupture with biliary peritonitis of hepatic hydatid cyst is an uncommon clinical presentation, even in endemic regions. It requires rupture both in the peritoneal cavity as well as in the biliary tree. Though intrabiliary rupture is the most common complication of hepatic hydatid cysts, intraperitoneal rupture is uncommon with an incidence ranging from 1% to 8% [1,2]. Combination of both complications is unusual. Presentation is usually dramatic with acute abdominal signs, such as guarding, rebound, and tenderness, are generally present. This complication should be included in the differential diagnosis of acute abdomen, especially in the endemic areas. Herein we report a case of biliary peritonitis due to spontaneous rupture of hydatid cyst of the right lobe of liver who presented with an acute abdomen.

## Case presentation

**Figure 1.**
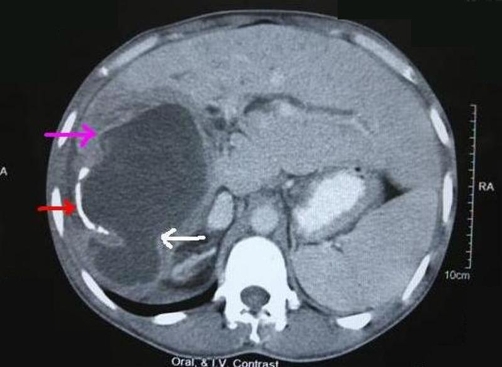
CECT abdomen showing a large hydatid cyst in the right lobe of the liver with wall calcification (red arrow) and separation of membranes (pink arrow). The endocyst has herniated through the ectocyst (white arrow), giving rise to a dumbbell shaped appearance.

**Figure 2.**
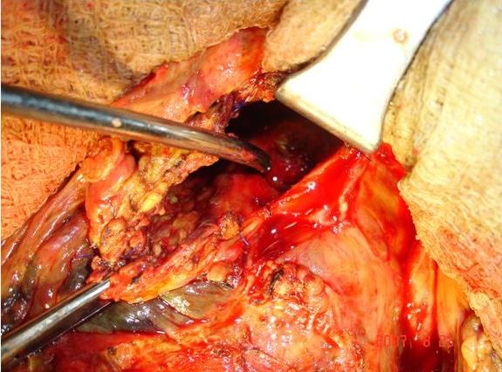
Operative photograph showing hydatid cyst cavity with bile tinge around the cavity. A metal dilator was introduced into the opening of bile duct in the cyst cavity.

A 34-year-old Indo-Aryan male of Indian nationality, previously fit and healthy presented with 15 days history of epigastric pain, jaundice and 7 days history of fever. He was initially evaluated at a local hospital with ultrasound and CT scan of the abdomen and was treated with parenteral antibiotics. He came to our emergency department for sudden increase in the intensity of abdominal pain and vomiting over past 8 hours. On examination he looked unwell and sweaty, with a temperature of 38.5°C and a pulse rate of 120 beats/min. The abdomen was tender with localized peritonitis in the right upper quadrant. Blood investigations revealed total count of 16400/mm^3^, total bilirubin of 7.1 mg/dl (conjugated-5.8 mg/dl), and alkaline phosphatase 701 U/L (normal range-60 to 120 U/L). Hydatid serology was positive. CT scan abdomen 2 days prior to admission to our institution showed a large hydatid cyst in the posterior segment of right lobe of liver with separation of membrane. It looked dumbbell shaped (Figure 1) and there was perihepatic fluid. The patient underwent emergent operation with a tentative diagnosis of intraperitoneal rupture of hydatid cyst. At laparotomy, 1.5L of bile stained fluid and multiple daughter cysts were found in the peritoneal cavity. A large hydatid cyst occupying parts of segments VI, VII, and VIII was seen (Figure 2). A rupture of the anterior wall of the pericyst, ectocyst and the endocyst was noted. The fluid was suctioned, daughter cysts removed, the anterior wall of the cyst deroofed and the endocyst removed. The cyst was then marsupialized with a 3-0 polydioxanone running suture. There was large defect in the intrahepatic bile duct and extrahepatic biliary tree was studded with hydatid debris. Cholecystectomy, CBD clearance and T tube drainage of CBD were done. Direct repair of intrahepatic bile duct defect was not possible. We introduced a 12 Fr T tube into the defect of intrahepatic bile duct and brought out through the skin. Two closed system drains were placed, one in the cyst cavity and one in the right subhepatic space. A thorough washout of cyst and peritoneal cavity was performed with both cetrimide and normal saline. Postoperatively, he developed biliary fistula, which was healed over a period of 34 days. T tube cholangiography on 42 days showed free flow of contrast in the duodenum and there was no extravasation of contrast or filling defect. The patient received a 6-week course of albendazole therapy in the postoperative period. At 4 months follow up, patient was without evidence of recurrence.

## Discussion

The organism Ecchinococcus granulosus causes hydatid disease, most commonly in the liver. It is endemic in sheep farming areas such as South and Central America, Western Europe, the Middle East, some sub-Saharan countries, Russia and China. In the life cycle, humans are inadvertent intermediate hosts, usually infected by direct contact with dogs or sheep [3].

Symptomatic hydatid cyst of liver may present with uncomplicated disease like hepatomegaly, right upper quadrant pain or acute complications. In about one third of the patients, cyst expansion may rupture into the biliary tree, erode through the diaphragm into the pleural cavity or rarely rupture into the peritoneal cavity. Intraperitoneal rupture is a rare but serious complication. According to Lewall and McCorkell [4], there are 3 types of cyst rupture: contained, communicating, and direct. Various incidence rates of direct rupture have been reported. While Sozuer *et al.* [1] reported a rate of 8.6%, Beyrouti *et al.* [2] reported an incidence rate of 1.75%. Rupture can occur spontaneously or following a trauma. The risk of rupture is reported to increase with the increased size of the cyst and intracystic pressure [5]. The clinical signs and symptoms of hydatid cyst rupture are not always severe, but in free perforation, hydatid fluid can cause chemical peritonitis Furthermore, peritoneal signs and symptoms may develop earlier and can be more severe if there is associated bile leak as occurred in our patient. Ultrasound and CT scan may be helpful for defining the cysts with detached membrane and the presence of intraabdominal fluid. Diagnostic peritoneal lavage is also helpful and is a highly specific test for hydatid cyst perforation. Treatment should be done expeditiously. Surgery continues to be the treatment of choice for perforated hydatid cysts. However, the best approach has not been clearly defined. Surgical methods used in the treatment of hydatid disease are radical (pericystectomy and hepatic resection) and conservative (unroofing associated with various procedures for the management of the residual cavity) procedures [6]. Radical procedures for such patients who need emergent operation may not always be feasible. In the study of Gunay *et al.* [7], only patients who were fit and could tolerate a radical procedure underwent such surgical procedures. On the contrary, conservative approaches are easy to perform, faster, and safer, and thus more appropriate for patients requiring urgent treatment [8]. All cyst contents should be removed. The cyst and entire peritoneal cavity should be lavaged with scolicidal agent and normal saline. Cholecystectomy and CBD exploration are done in presence of obstructive jaundice. Cystobiliary communications are dealt according to the site and size of the fistulae. Direct suturing is contraindicated in large, lateral, or central fistulae. In such cases, a catheter is introduced through the fistula orifice and is left in the intrahepatic biliary tree and is brought out through the skin along the shortest route similar to our case. Others advocate cystojejunostomy. Overall, perioperative complications are reported to be as high as 50%, though the recurrence rates are not as high as once thought [7]. All patients should receive albendazole for at least 1 month to reduce recurrence rate. Follow up of patients with imaging and serology at 6 months interval is justified to detect recurrence.

## Conclusion

Though rare, intraperitoneal rupture of hydatid cyst should be considered in the differential diagnosis of acute abdomen particularly in persons residing in the endemic zone of hydatid cyst. If the patient is jaundiced and preoperative imaging study is suggestive of hydatid cyst, one should keep in mind the possibility of biliary peritonitis as a cause of acute abdomen.

## Abbreviations

CBD: common bile duct
CT: computed tomography
Fr: French gauge
